# Accurate Assignment of Significance to Neuropeptide Identifications Using Monte Carlo K-Permuted Decoy Databases

**DOI:** 10.1371/journal.pone.0111112

**Published:** 2014-10-17

**Authors:** Malik N. Akhtar, Bruce R. Southey, Per E. Andrén, Jonathan V. Sweedler, Sandra L. Rodriguez-Zas

**Affiliations:** 1 Department of Animal Sciences, University of Illinois at Urbana-Champaign, Urbana, Illinois, United States of America; 2 Department of Pharmaceutical Biosciences, Uppsala University, Uppsala, Sweden; 3 Department of Chemistry, University of Illinois at Urbana-Champaign, Urbana, Illinois, United States of America; 4 Department of Statistics, University of Illinois at Urbana-Champaign, Urbana, Illinois, United States of America; 5 Institute for Genomic Biology, University of Illinois at Urbana-Champaign, Urbana, Illinois, United States of America; Swiss Institute of Bioinformatics, Switzerland

## Abstract

In support of accurate neuropeptide identification in mass spectrometry experiments, novel Monte Carlo permutation testing was used to compute significance values. Testing was based on k-permuted decoy databases, where k denotes the number of permutations. These databases were integrated with a range of peptide identification indicators from three popular open-source database search software (OMSSA, Crux, and X! Tandem) to assess the statistical significance of neuropeptide spectra matches. Significance *p-values* were computed as the fraction of the sequences in the database with match indicator value better than or equal to the true target spectra. When applied to a test-bed of all known manually annotated mouse neuropeptides, permutation tests with k-permuted decoy databases identified up to 100% of the neuropeptides at *p-value* < 10^−5^. The permutation test *p-values* using hyperscore (X! Tandem), *E-value* (OMSSA) and Sp score (Crux) match indicators outperformed all other match indicators. The robust performance to detect peptides of the intuitive indicator “number of matched ions between the experimental and theoretical spectra” highlights the importance of considering this indicator when the *p-value* was borderline significant. Our findings suggest permutation decoy databases of size 1×10^5^ are adequate to accurately detect neuropeptides and this can be exploited to increase the speed of the search. The straightforward Monte Carlo permutation testing (comparable to a zero order Markov model) can be easily combined with existing peptide identification software to enable accurate and effective neuropeptide detection. The source code is available at http://stagbeetle.animal.uiuc.edu/pepshop/MSMSpermutationtesting.

## Introduction

Neuropeptides participate in cell to cell communication and regulate many biological processes such as behavior, learning, and metabolism [1]. Mass spectrometry has revolutionized neuropeptide characterization and quantification [2]–[7]. However, detection is complicated by the neuropeptide size (typically 3 to 40 amino acids long) and by the complex post-translational processing that includes cleavage, and amino acid modifications of prohormones into neuropeptides [1], [8].

Database search programs are commonly used to identify peptides from tandem mass spectrometry experiments [9]. These programs generate *in silico* theoretical spectra from target databases of known peptide sequences that have masses within a range (tolerance) of the observed peptide mass. The *in silico* spectra are then compared to the observed experimental spectra and indicator scores that signify the closeness of the match are computed. To assess the statistical significance of these matches, the observed-target match indicator is compared to the distribution of indicator values under the null hypothesis of no match using various methods. In the popular target-decoy approach, the experimental spectra are compared to spectra from a decoy database consisting of peptides sequences that were generated by reverting or reshuffling the amino acids in the sequences of the target database [9]–[11].

For neuropeptide identification, the target-decoy approach can result in false negatives because the small size of many neuropeptides leads to low observed-target match indicator values and consequently low significance levels [11]. Furthermore, the small size of many neuropeptide leads to few decoy reshuffled sequences and the resulting granularity of the null distribution of decoy scores further lowers the significance levels [11]–[15]. At the protein level, alternative identification approaches have attempted to address the challenge of assessing statistical significance [16], [17]. However, the implementations of the previous approaches do not work with widely used database search programs, do not use all the information resulting from the mass spectrometry experiment, and are biased by peptide length or assume one-direction progressive processing. Approaches that rely on fewer limiting assumptions and that use all the information available need to be evaluated.

Permutation tests are well-suited for neuropeptide database searches by helping to overcome the finite combination of amino acids from small neuropeptides and do not rely on directional assumptions. Furthermore, permutation testing provides strong control of Type I errors thus minimizing the incidence of false positive results [18]. Under the null hypothesis of no match, the experimental spectrum of a peptide is the result of a random sequence of amino acids provided that the total mass is close to the experimental mass. This requirement stems from the database search program strategy that only accepts sequences within a user determined range of the experimental spectra. Computation of the permutation statistical significance requires the distribution of the peptide-spectrum match scores generated by the database search program under the null hypothesis that there is no correct match. This distribution is then generated by searching the experimental spectrum against a decoy database considering all possible amino acid sequences within the predetermined range of the experimental spectra. The permutation *p-value* is then the proportion of peptide-spectrum scores obtained from the decoy database that are equal to greater to the score obtained using the target database. Under the null hypothesis any amino acid can be present at any position of the sequence, thus, addressing the exchangeable assumption required by the permutation test [18].

Monte Carlo sampling is used to reduce the number of possible sequences while providing an unbiased estimate of the *p-value*. Furthermore, the loss in statistical efficiency when estimating the *p-value* decreases with increasing number of random samples [18]. The previously demonstrated advantage of the Monte Carlo permutation approach proposed over existing decoy generation based on sequence reversion or reshuffling of the target sequence is the improved definition of the null distribution [18]. The larger number of decoy sequences results in lower granularity and, thus, more precise assessment of the statistical significance of the observed matches. Two major advantages of the Monte Carlo permutation approach proposed over existing dynamic programming approaches [16], [17] is the simplicity of integration to existing database search programs, the use of all spectra information available and consideration of all possible spectra matching processes.

This study demonstrates the use Monte Carlo permutation testing to overcome the limitations of current protein identification approaches to accurately assess neuropeptide statistical significance. This approach combines and extends the model-free property of current decoy databases with the more extensive search of dynamic programming approaches. The aims are: (1) to develop permutation resampling methodology that can be easily integrated with existing peptide database search software, and (2) to demonstrate the advantages of this approach to provide accurate measures of neuropeptide match significance using ideal and real experimental neuropeptide spectra. Supporting objectives were: (1) to develop and implement complementary novel permuted databases; (2) to determine the number of permutations required for accurate significance levels; and (3) to identify the neuropeptide match indicators within and across programs that are better suited to provide accurate statistical significance.

## Materials and Methods

### Tandem Spectral Dataset and Target Database

Tandem mass spectra from a comprehensive list of 103 experimentally-obtained and manually annotated mouse neuropeptide were obtained from the SwePep database (http://www.swepep.org). These spectra were obtained using linear ion trap mass spectrometer coupled with liquid chromatography and electrospray ionization source [19]. Neuropeptides were manually validated after identification using the X! Tandem database search program [20]. The independent manual annotation step also ensured that the subsequent software comparison would not be biased in favor of the X! Tandem database search program. Of these, 80 neuropeptides were unmodified and the remaining 23 encompassed post-translational modifications (PTMs) including C-terminal amidation, N-terminal acetylation, phosphorylation, pyroglutamination and oxidation. The spectra corresponded to 5, 68, 25, and 5 peptides that had precursor charge states +1, +2, +3 and +4, respectively, and all charge states were observed in modified and unmodified peptides.

Ideal uniform spectra of all possible *b*- and *y*-ions with +1 product charge state were simulated for 103 annotated experimental spectra. The ideal spectra also included all the PTMs identified in the corresponding experimental spectra. The neutral mass loss peaks due to loss of single water or ammonia molecules from the *b*- and *y*-ions were simulated regardless of their position in the ions sequence. These ideal spectra are expected to be correctly identified at an extremely high significance level because these spectra are equivalent to the theoretical spectra internally generated by the database search engine.

A comprehensive target database of 618 mouse neuropeptides was obtained from the PepShop database (http://stagbeetle.animal.uiuc.edu/pepshop; [21]). This target database encompassed the neuropeptides corresponding to the 103 tandem spectra studied. The neuropeptides in the PepShop were assembled from the known 95 mouse prohormones present in SwePep [19] and UniProt [22] complemented with NeuroPred [23] predictions. The neuropeptides in the target database ranged from 2 to 223 amino acids in length because this included all known experimentally confirmed mouse neuropeptides as well as all possible intermediate and other peptides produced during the processing of prohormones. The target database of neuropeptides is available at http://stagbeetle.animal.uiuc.edu/pepshop/MSMSpermutationtesting.

### Database Search Programs and Database Searching

Three open source database search programs were used in this study: Crux [24] (version 1.37), OMSSA [25] (version 2.1.8), and X! Tandem [20] (version 2013.02.01.1). These commonly used open source programs were selected because the code could be modified to ensure comparable search parameter specification and enabled to retrieve intermediate indicators of the strength of the match between the observed and target or decoy spectra. The observed-target or observed-decoy spectra match indicators extracted from OMSSA were: number of matched fragment ions, lambda or Poisson mean match indicator, Poisson probability of the lambda match indicator, and corresponding *E-value* of the match (Poisson probability multiplied by the effective database size). The spectra match indicators extracted from X! Tandem were: number of matched fragment ions, intermediate convolution score (product of the intensities of the shared *b*- and *y*-fragment ions between experimental and theoretical spectra), hyperscore (factorial of the number of matching *b*- and *y*-ions multiplied by the convolution score), and *E*-*value* (calculated from the distribution of hyperscores scores). The spectra match indicators extracted from Crux were: number of matched fragment ions, Sequest Sp score (Sp), cross-correlation score (XCorr), deltaCn score (ΔCn) and *p-value* that is calculated from the Weibull distribution fitted to the XCorr scores of observed-theoretical spectra matches [26].

For comparable neuropeptide identification across the three programs the following search parameters from our prior research [11] were used: (1) precursor ion tolerance: 1.5 Da; (2) fragment ion tolerance: 0.3 Da (OMSSA and X! Tandem); mz-bin-width: 0.3 (Crux) (3) searches were performed with and without PTMs. The PTMs evaluated were: amidation, phosphorylation, N-terminal acetylation, acetylation of lysine, pyroglutamination of glutamine, methylation of lysine and arginine residues, sulfation of tyrosine residue, and oxidation of methionine; (4) “protein” (OMSSA) or “enzyme: custom cleavage site” (X! Tandem and Crux) to prevent peptide cleavage since the detection of neuropeptides does not involve protease digestion; (5) fragment ion charge: default values; (6) OMSSA “ht” option was set to eight to filter database peptides that have at-least one theoretical fragment ion match to one of the top eight most intense peaks in the observed spectra; and (7) peptide mass: monoisotopic; 8) Crux *p-values* were computed using 1000 Weibull points because this information provides more accurate *p-values* than the default 40 Weibull points [11].

### Permutation Approach and K-Permuted Decoy Databases

**Figure 1:**
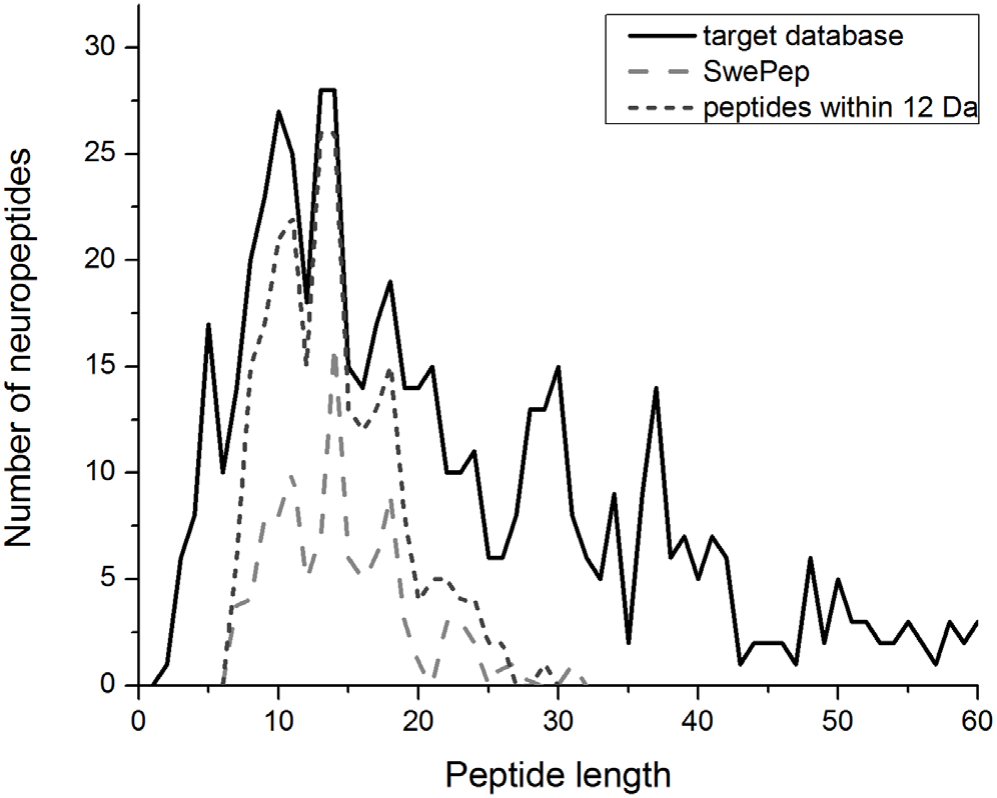
Distribution of neuropeptides length in target database peptides (less than 60 amino acid in length are shown), 103 studied peptides, and 236 peptides that fall within ±12 Da of the 103 peptides.

A Monte Carlo permutation test approach based on biological, computational and statistical considerations was used to generate decoy sequence databases. The resulting decoy database accommodates limited changes in the specified search parameters and can be used by all database search programs without the need to modify the original program code. A decoy database considering all possible amino acid sequences can be used to generate the distribution of the peptide-spectrum match scores under the null hypothesis of no correct match. This distribution is required by database search programs to assess the statistical significance of the match. This requirement results in an extremely large number of sequence since a decoy database of only 10-amino acid long peptides consists of 6.13×10^12^ sequences. This number would be further increased to account for different possible lengths of the target neuropeptides. Due to the potential size of a database encompassing all possible sequences, a Monte Carlo permutation approach based on a set of candidate peptides was used to generate a random sample of all possible sequences. Applying the same strategy used by the database search programs, 236 mouse neuropeptides within 12 Da of the precursor mass of the 103 studied neuropeptides were considered as candidate peptides. The 12 Da arbitrary threshold enabled the creation of single flexible peptide catalog that could be used with different database programs while permitting different mass tolerance specifications and precursor charge states. The arbitrary threshold does not influence the results because the database search programs ignore candidate sequences outside the settings and all peptide-spectrum scores involving target database size will be equally affected. Figure 1 depicts the correspondence between the lengths of neuropeptides in the target database, the 103 experimental neuropeptides and the neuropeptides that fall within 12 Da of the 103 peptides. A new decoy peptide database was obtained by generating a set of random sequences from each of the candidate peptides. Random sequences were generated by sequentially replacing each amino acid in the sequence of each candidate peptide by a randomly selected amino acid from the 19 amino acids from the candidate peptide list (leucine and isoleucine were considered the same amino acid due to the same neutral masses). This was repeated until the predetermined number of permuted sequences per candidate sequence was obtained. The resulting permuted sequences are comparable to those generated from a Markov model of order zero such that the 19 amino acids are equally likely at all positions. These permuted sequences were collected into a single database after removal of duplicate peptides and sequences present in the target database. This procedure was used to generate k-permuted decoy sequence databases where the numbers of unique permuted sequences per candidate peptide (k) were: 10^3^ (K10^3^ with 236,000 decoy peptide sequences), 10^4^ (K10^4^ with 2,360,000 decoy peptide sequences), 10^5^ (K10^5^ with 23,600,000 decoy peptide sequences), and 10^6^ (K10^6^ with 236,000,000 decoy peptide sequences). The target database was appended to each of the four k-permuted databases to create a combined target-k-permuted decoy database. The combined database search is more accurate than separate database searches and to avoid zero *p-value* [27]–[29]. This strategy also removed potential database size dependency of the match indicators between target and permuted sequences because the correct match was evaluated under the same database sizes as the permuted databases.

**Figure 2:**
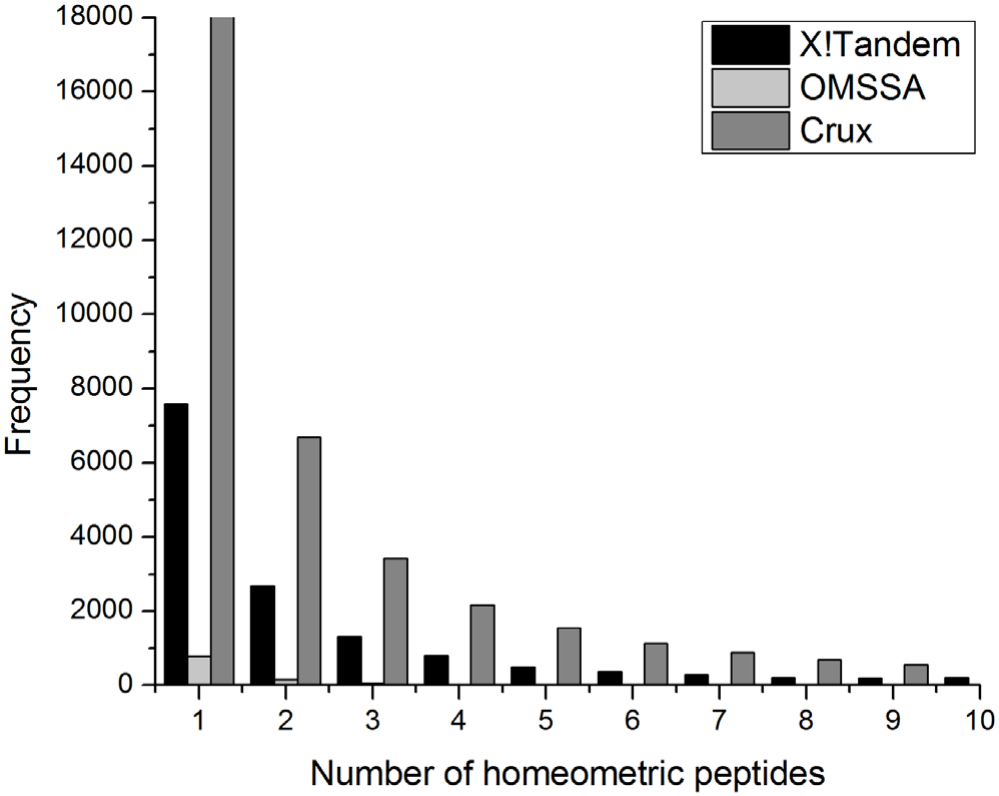
Frequency (number) of spectra with 1 to 10 homeometric matches for K10^6 ^k-permuted decoy databases across the three database search programs (X! Tandem, OMSSA, and Crux).

The search of spectra against the k-permuted decoy databases produced many matches that were indistinguishable from each other based on the indicators reported by the programs (e.g., number of matched ions, hyperscore, convolution score, and *E-value* for the X! Tandem). Matches were considered “homeometric” [18] when the matches had the same indicator values across programs and the matched peptides masses were within ±1.5 Da from each other. Figure 2 depicts the number of peptides with homeometric matches ranging from 1 to 10 for the K10^6 ^k-permuted decoy database across the three databases search programs. Homeometric matches were counted only once while calculating the number of random peptides that have an indicator value equal or better than the true target peptide. This strategy resolved the challenge that database search programs were not able to differentiate between such matches that are technically redundant and ensured the calculation of permutation *p-values* that were unbiased by these effects.

For each database search program and target sequence, the observed tandem spectra were searched for matches within each combined target-k-permuted decoy spectra. The permutation *p-values* were estimated as the fraction of combined target-k-permuted decoy peptides, excluding any homeometric matches that have a matching indicator score equal or better than the score of target peptide.

A comprehensive evaluation of the k-permuted decoy approaches, programs, and peptide match indicators was undertaken including: (a) Search for ideal uniform simulated spectra against the target database using all three database search programs; (b) Search for real tandem spectra against the target database using all three database search programs; (c) Search for the 80 tandem spectra containing no PTMs against the K10^3^, K10^4^, K10^5^, and K10^6^ target-k-permuted decoy databases without PTM specification using all three database search programs; (d) Search for the 80 tandem spectra containing no PTMs against the K10^5 ^k-permuted database with PTM specification using all three database search programs; and (e) Search for the 23 tandem spectra containing PTMs against the K10^5 ^k-permuted database with PTM specification using OMSSA and X! Tandem. Crux was excluded from this last comparison due to considerable amount of search time required.

## Results and Discussion

A k-permuted decoy database approach that resolves limitations of existing methods to assess the significance of peptides is presented. The proposed approach can be used with any database search program, especially when the program lacks of a statistical approach for *p-value* calculation, and can be integrated to target and target-decoy databases. The k-permutation strategy proposed supersedes a decoy database of randomly generated decoy peptides that underestimates the false discovery rate because, by definition, the vast majority of the random peptides will be true negatives. Non-random approaches such as reversing peptide sequences are more appropriate than random approaches in generating decoy databases [9]–[11].

Results from a three step benchmarking strategy were used to evaluate the performance to detect neuropeptides using target-k-permuted decoy databases. First, a baseline performance was obtained by comparing ideal simulated spectra against a standard “target database” using the three database search programs. Then, observed tandem spectra were matched to a target database. Lastly, the observed tandem spectra were matched to different target-k-permuted decoy databases. Evaluation of results among modified and non-modified spectra enabled understanding of the influence of PTMs on the search results. The source code to generate k-permuted decoy databases is available at http://stagbeetle.animal.uiuc.edu/pepshop/MSMSpermutationtesting.

### Peptide Detection using Ideal Simulated Spectra and a Target Database

Table 1 summarizes the results from the three database search programs when 103 ideal uniform spectra were simulated with all *b*- and *y*-ions including neutral mass losses and searched against the target database. The search of ideal simulated spectra demonstrated the ability of the database search methods to assign an E*-value* or *p-value* to each peptide-spectrum match in the absence of technical or biological noise [30]. Although the E-*values* and *p-values* reported by these programs have different interpretations and are computed differently between programs, match counts based on the same threshold are reported to facilitate the identification of trends.

**Table 1:** Peptide detection significance levels using ideal simulated spectra of the 103 peptides with and without any post-translational modifications (PTMs) and all *b*- and *y*-ions including neutral mass losses against a standard target database across database search programs (OMSSA, X! Tandem, and Crux).

Program	PTMs	Significance^a^							P≤10^−2b^
		0	1	2	3	4	5	≥6	
X! Tandem	None	0	0	4	4	2	6	58	74
	Amidation	0	0	0	0	0	0	9	9
	Oxidation	0	0	0	0	0	0	1	1
	Pyroglutamination	0	0	1	0	1	1	1	4
	Phosphorylation	0	0	0	0	0	1	3	4
	N-terminal acetylation	0	0	0	0	0	0	0	0
OMSSA	None	0	0	0	0	0	0	79	79
	Amidation	0	0	0	0	0	0	9	9
	Oxidation	0	0	0	0	0	0	1	1
	Pyroglutamination	0	0	1	2	1	0	0	4
	Phosphorylation	0	0	0	0	0	0	4	4
	N-terminal acetylation	0	0	0	0	0	0	5	5
Crux	None	2	5	12	52	3	1	2	70
	Amidation	0	0	2	3	2	0	2	9
	Oxidation	0	0	0	1	0	0	0	1
	Pyroglutamination	0	1	1	0	1	0	1	3
	Phosphorylation	0	0	1	2	0	0	1	4
	N-terminal acetylation	0	0	0	3	1	0	1	5

a Significance threshold (t) for matched to be considered significant at an E*-value* or *p-value* < 1×10^−t^ (t = 0 to > = 6).

b Cumulative number of peptides with an E*-value* or *p-value* < 1×10^−2^.

The three programs matched all unmodified neuropeptides correctly at an *E-value* or *p-value* < 2×10^−1^. At an E*-value* or *p-value* < 1×10^−2^, OMSSA, X! Tandem, and Crux identified 80 (100% of unmodified neuropeptides), 80 (100% of unmodified neuropeptides), and 73 (91.25% of unmodified neuropeptides) peptides, respectively. This trend was consistent with previous study that compared Crux, OMSSA and X! Tandem [11]. Our study confirmed the lower significance values that Crux computes for peptides less than 45 amino acids in length [11]. OMSSA *E-values* averaged more significant matches than X! Tandem for the 32 peptides that were less than 13 amino acids in length. However, for the 48 peptides longer than 12 amino acids in length, the difference in significance levels of X! Tandem and OMSSA decreased on the average with 8, 18, and 22 peptides getting lower, equal, and better significance levels for the X! Tandem than OMSSA, respectively.

For the 23 neuropeptides with PTMs and an E*-value or p-value* < 1×10^−1^, OMSSA, X! Tandem, and Crux correctly detected 23 (100% of modified neuropeptides), 18 (78.26% of modified neuropeptides), and 23 (100% of modified neuropeptides) peptides, respectively. X! Tandem failed to correctly match five peptides with N-terminal acetylation modification instead these five peptides were matched with incorrect internal acetylation modification at 9^th^ lysine residue. The failure in the peptide detection of X! Tandem was only observed when multiple PTMs were specified in the search specification. The five peptides were correctly detected when only N-terminal acetylation was used in the search specification. At an *E-value* or *p-value* < 1×10^−2^, 23 (100% of modified neuropeptides), 18 (78.26% of modified neuropeptides), and 22 (95.66% of modified neuropeptides) peptides were detected by OMSSA, X! Tandem, and Crux, respectively. The three peptides that were not significant for OMSSA at *E-value*<1×10^−4^ all had a pyroglutamination (Q residue) modification. Two of these peptides, somatostatin (gene symbol: SMS) [87–100] (QRSANSNPAMAPRE; charge state +2) and secretogranin-2 (gene symbol: SCG2) [205–216] (QELGKLTGPSNQ; charge state +1), were significant for the X! Tandem and Crux at an E*-value* or *p-value* < 1×10^−4^. A nine amino acid long peptide secretogranin-1 (gene symbol: SCG1) [667–675] (QKIAEKFSQ; charge state +2) was not significant for all three programs at an *E-value* or *p-value* < 1×10^−4^, while the same peptide was missed by the Crux at *p-value* < 1×10^−2^.

### Peptide Detection using Observed Spectra and a Target Database

Table 2 summarizes the performance of the three database search programs when the 80 experimental tandem spectra containing no PTMs were searched against the target database. All peptide assignments by the three database search methods were correct at an *E-value* or *p-value* < 5×10^−1^. At an *E-value* or *p-value* < 1×10^−2^, OMSSA, X! Tandem and Crux detected 80 (100% of unmodified neuropeptides), 71 (88.75% of unmodified neuropeptides), and 63 (78.75% of unmodified neuropeptides) peptides, respectively. The higher number of significant peptide detections by OMSSA relative to Crux was consistent with the prior reports [11]. The three search methods were less accurate on 23 observed spectra with PTMs when searched against the standard target database (Table 2). From the correctly matched peptides for each program, at an *E-value* or *p-value* < 1×10^−2^, OMSSA, X! Tandem and Crux detected 20 (86.95% of modified neuropeptides), 15 (65.21% of modified neuropeptides), and 17 (73.91% of modified neuropeptides) peptides, respectively.

**Table 2:** Peptide detection significance levels using experimental spectra of the 103 peptides with and without any post-translational modifications (PTMs) against a standard target database across database search programs (OMSSA, X! Tandem, and Crux).

Program	PTMs			Significance^a^							Cum N^b^
		Miss^c^	Inc^d^	0	1	2	3	4	5	≥6	P≤10^−2^
X! Tandem	None	0	0	1	8	11	15	16	11	18	71
	Amidation	0	0	0	0	0	0	0	0	9	9
	Oxidation	0	0	0	0	0	0	0	0	1	1
	Pyroglutamination	0	0	0	0	1	0	1	1	1	4
	Phosphorylation	0	0	0	0	0	0	0	1	3	4
	N-terminal acetylation	0	5	0	0	0	0	0	0	0	0
OMSSA	None	0	0	0	0	1	2	1	3	73	80
	Amidation	1	0	1	0	0	0	1	0	6	7
	Oxidation	0	0	0	0	0	0	0	0	1	1
	Pyroglutamination	0	0	0	0	0	0	1	0	3	4
	Phosphorylation	0	0	0	0	0	0	0	0	4	4
	N-terminal acetylation	0	1	0	0	0	0	0	0	4	4
Crux	None	0	0	9	8	9	44	1	0	9	63
	Amidation	0	0	0	1	5	1	1	0	1	8
	Oxidation	0	0	0	0	0	1	0	0	0	1
	Pyroglutamination	0	0	1	1	0	2	0	0	0	2
	Phosphorylation	0	0	0	2	2	0	0	0	0	2
	N-terminal acetylation	0	0	0	1	1	2	0	1	0	4

a Significance threshold (t) for matched to be considered significant at an E*-value* or *p-value* < 1×10^−t^ (t = 0 to > = 6).

b Cumulative number of peptides with an E*-value* or *p-value* < 1×10^−2^.

c Number of peptides missed by program.

d Number of peptides with incorrect post-translational modification assignment.

The 80 spectra without PTMs were searched against the target database using three database search programs and with PTM specifications. X! Tandem peptide detection significance levels for the 76, 3, and 1 target peptide remained unchanged, decreased, and increased, respectively, relative to the searches involving no PTMs. The changes in the significance levels of the four peptides were due to higher number of candidate peptides available in the PTM searches which in turn changed the estimation parameters used in the *E-value* computation. The OMSSA peptide detection significance levels decreased for the majority of the previous peptides (75 out of 80 peptides) or remained unchanged (5 out of 80 peptides) when searches included PTMs, respectively. Crux peptide detection significance levels were improved when searches included PTMs with 65 and 29 peptide detections at *p-value* < 1×10^−2^ and <1×10^−4^, respectively. Comparison of peptide detections across PTM scenarios indicated that at *p-value* < 1×10^−2^, 54 peptides were detected by both scenarios, 11 peptides were detected in the PTM scenario, 9 peptides were detected in the no PTMs scenario, and 6 peptides were not detected by either scenario. The target peptides with low XCorr scores remained undetected either across both scenarios or with PTM search. The clear positive correlation between significance level and XCorr score for the PTM searches relative to the searches without PTMs could be due to the higher number of low scoring matches in the searches with PTMs than without PTMs. The Crux resampling from the low scoring matches might have resulted in a shift on the distribution of XCorr scores towards lower scores than the target peptides XCorr scores.

### X! Tandem Peptide Identification using a K-Permuted Decoy Database

Table 3 summarizes the log_10_ transformed of the *E-values* to the target database and permutation *p-values* computed for the X! Tandem indicators: number of matched ions, hyperscore, *E-value*, and convolution score using the 80 spectra without PTMs across the four target-k-permuted decoy databases studied. The permutation *p-values* from number of matched ions, hyperscore and *E-value* showed that the X! Tandem *E-values* from the target database were dramatically underestimated (less significant) for most target peptides. Detection and significance level using the number of ions matched, hyperscore and *E-value* were almost the same across all target-k-permuted decoy databases. Only at the 10^6^ permutations did the *p-values* for number of ions matched started to differ from the *p-values* from the hyperscore and *E-value* match indicators. This trend was expected as the hyperscore is a function of the product of factorial of the number of matched ions and the ion intensity values and *E-value* is a function of the hyperscore.

**Table 3:** Performance of the target and alternative k-permuted decoy databases used with the X! Tandem database search program using spectra from 80 unmodified neuropeptides.

Database^a^	Indicator	Significance Levels of the Permutation *p-values* b							Cum. Num. of Peptides^c^	
		0	1	2	3	4	5	≥6	≥10^−2^	≥10^−4^
Target	*E-value*	1	8	11	15	16	12	17	71	45
K10^3^	# ions	0	0	76	4	0	0	0	80	0
	Hyperscore	0	0	76	4	0	0	0	80	0
	Convolution	0	8	70	2	0	0	0	72	0
	*E-value*	0	0	76	4	0	0	0	80	0
K10^4^	# ions	0	0	0	80	0	0	0	80	0
	Hyperscore	0	0	0	80	0	0	0	80	0
	Convolution	0	5	44	31	0	0	0	75	0
	*E-value*	0	0	0	80	0	0	0	80	0
K10^5^	# ions	0	0	0	0	80	0	0	80	80
	Hyperscore	0	0	0	0	80	0	0	80	80
	Convolution	0	3	36	32	9	0	0	77	9
	*E-value*	0	0	0	0	80	0	0	80	80
K10^6^	# ions	0	0	0	0	1	79	0	80	80
	Hyperscore	0	0	0	0	0	80	0	80	80
	Convolution	0	4	30	36	5	5	0	76	10
	*E-value*	0	0	0	0	0	80	0	80	80

a Target: database of 236 neuropeptide sequences; K10^3^: k-permuted decoy database size of 236,000 peptides; K10^4^: k-permuted decoy database size = 2,360,000 peptides; K10^5^: k-permuted decoy database size = 23,600,000 peptides; K10^6^: k-permuted decoy database size = 236,000,000 peptides.

b Significance threshold (t) for target spectrum to be considered significant at significance thresholds <1×10^−t^ (t = 0 to > = 6).

c The cumulative number of peptides at 1×10^−2^ and 1×10^−4^ thresholds.

The convolution score resulted in fewer target peptide identifications with higher number of sequence permutations due to relative increase in the number of decoy matches with equal or better scores. From the K10^3^, K10^4^, K10^5^, and K10^6^ target-k-permuted decoy databases, 72 (90% of unmodified neuropeptides), 31 (39% of unmodified neuropeptides), 9 (11% of unmodified neuropeptides), and 10 (13% of unmodified neuropeptides) peptides were identified at *p-value* < 1×10^−2^, <1×10^−3^, <1×10^−4^, and <1×10^−4^, respectively. These results showed that the convolution score alone was less suitable to discriminate between true target and decoy matches than the hyperscore and *E-value*.

Comparison of the *p-values* obtained from the target-k-permuted decoy number of matched ions, hyperscores and convolution scores suggested that roughly 10^5^ permutations were required for significant *p-value* computations using the convolution scores. Higher number of sequence permutations provided better separation between the significance levels of the three indicators. There were 7 peptides with *E-values*<10^−7^ from the target database indicating that the lower bound of *p-values* appeared to be far smaller than the limit provided by the K10^6^ permuted database. Comparable performance (significance level) using number of matched ions and hyperscore were observed with fewer permutations or lower significance thresholds. This novel finding suggests that more significant detections can be obtained by permuting the X! Tandem hyperscore and number of matched ions indicators, even with a relatively small k-permuted decoy database size.

### Crux Peptide Identification using a K-Permuted Decoy Database

Table 4 summarizes the log_10_ transformed permutation *p-values* computed for the Crux match indicators: number of matched ions, XCorr, ΔCn, and Sp using the 80 spectra without PTMs across the four target-k-permuted decoy databases. Higher number of sequence permutations increased the significance values using the number of matched ions and Sp. This trend was due to the lower number of matched ions and Sp scores of the decoy peptide matches relative to the target peptides. The two non-detected peptides could be attributed to the low number of decoy candidates for those peptides rather than to an increase in the number of decoy peptides with equal or better scores. The hindering effect on the match significance of better or equal decoy matches on Sp was more evident with the large decoy databases at *p-value* < 1×10^−5^.

**Table 4:** Performance of the target and alternative k-permuted decoy databases used with the Crux database search program using spectra from 80 unmodified neuropeptides.

Database^a^	Indicator^b^	Significance Levels of the Permutation *p-values* c							Cum. Num. of peptides^d^	
		**0**	**1**	**2**	**3**	**4**	**5**	**≥6**	**≥10^−2^**	**≥10^−4^**
Target	*p-value*	9	8	9	44	1	0	9	63	10
K10^3^	# ions	0	2	78	0	0	0	0	78	0
	XCorr	3	11	66	0	0	0	0	66	0
	Sp	0	2	78	0	0	0	0	78	0
	ΔCn	3	11	66	0	0	0	0	66	0
K10^4^	# ions	0	0	1	79	0	0	0	80	0
	XCorr	3	10	14	53	0	0	0	67	0
	Sp	0	0	1	79	0	0	0	80	0
	ΔCn	3	10	14	53	0	0	0	67	0
K10^5^	# ions	0	0	0	1	79	0	0	80	79
	XCorr	3	10	8	23	36	0	0	67	36
	Sp	0	0	0	1	79	0	0	80	79
	ΔCn	3	10	8	23	36	0	0	67	36
K10^6^	# ions	0	0	0	0	2	78	0	80	80
	XCorr	3	10	9	19	22	17	0	67	39
	Sp	0	0	0	0	4	76	0	80	80
	ΔCn	3	10	9	19	22	17	0	67	39

a Target: database of 236 neuropeptide sequences; K10^3^: k-permuted decoy database size of 236,000 peptides; K10^4^: k-permuted decoy database size = 2,360,000 peptides; K10^5^: k-permuted decoy database size = 23,600,000 peptides; K10^6^: k-permuted decoy database size = 236,000,000 peptides.

b # ions: permutation *p*-*values* computed for the number of matched *b*- and *y*-ions. XCorr: permutation *p*-*values* computed from the XCorr scores of the matches. Sp: permutation *p*-*values* computed from the Sp scores of the matches. ΔCn: permutation *p*-*values* computed using X! Tandem ΔCn.

c Significance threshold (t) for matched to be considered significant at *p*-*value*<1×10^−t^.

d Cumulative number of peptides with *p*-*values* thresholds of 1×10^−2^ and 1×10^−4^.

Peptide detection was less significant when using XCorr relative to Sp and number of matching ions. The drop in significance level with increase in threshold and database size was due to the higher number of decoy peptides reaching XCorr levels better or equal than the target peptides. The detection and significance computation using XCorr and ΔCn (the difference in XCorr between candidates) was similar across all target-k-permuted databases which reflects that the range of these match indicators stabilized. The range of possible XCorr values was limited by the number of observed spectrum peaks because the background adjustment is expected to be constant across permuted database sizes. This result indicates that only a relatively few permuted sequences are required to cover the range of XCorr values and that higher number of permutations offer greater precision to detect match differences.

### OMSSA Peptide Identification using a K-Permuted Decoy Database

Table 5 summarizes the log_10_ transformed permutation *p-values* calculated for the OMSSA match indicators: number of matched ions, lambda match indicator, *p-value*, and *E-value* using the 80 spectra without PTMs across the target-k-permuted decoy databases. Comparison between the target database and the permutation *p-values* indicated that most peptides were accurately estimated by OMSSA suggesting that the k-permuted database size was unimportant. Examination of the few peptides with underestimated *E-values* suggested that these peptides had fewer intense MS/MS ion peaks resulting in lower 75% quartile values than peptides of similar size with lower *E-values*. This result indicates that OMSSA *E-values* may be less reliable in the presence of multiple low intensity spectra peaks.

**Table 5:** Performance of the target alternative k-permuted decoy databases used with the OMSSA database search program using spectra from 80 unmodified neuropeptides.

Database^a^	Indicator^b^	Significance Levels of the Permutation *p-values* c							Cum. Num. of Peptides^d^	
		0	1	2	3	4	5	≥6	≥10^−2^	≥10^−4^
Target	*E-value*	0	0	1	2	1	3	73	80	77
K10^3^	# ions	0	2	78	0	0	0	0	78	0
	Lambda	0	9	71	0	0	0	0	71	0
	*p-value*	0	2	78	0	0	0	0	78	0
	*E-value*	0	2	78	0	0	0	0	78	0
K10^4^	# ions	0	0	1	79	0	0	0	80	0
	Lambda	0	5	11	64	0	0	0	75	0
	*p-value*	0	0	1	79	0	0	0	80	0
	*E-value*	0	0	1	79	0	0	0	80	0
K10^5^	# ions	0	0	0	0	80	0	0	80	80
	Lambda	0	5	8	24	43	0	0	75	43
	*p-value*	0	0	0	0	80	0	0	80	80
	*E-value*	0	0	0	0	80	0	0	80	80
K10^6^	# ions	0	0	0	0	2	78	0	80	80
	Lambda	0	5	8	17	18	32	0	75	50
	*p-value*	0	0	0	0	0	80	0	80	80
	*E-value*	0	0	0	0	0	80	0	80	80

a Target: database of 236 neuropeptide sequences; K10^3^: k-permuted decoy database size of 236,000 peptides; K10^4^: k-permuted decoy database size = 2,360,000 peptides; K10^5^: k-permuted decoy database size = 23,600,000 peptides; K10^6^: k-permuted decoy database size = 236,000,000 peptides.

b # ions: permutation *p*-*values* computed for the number of matched *b*- and *y*-ions. Lambda: permutation *p*-*values* computed from the Poisson mean of matches. *p*-*value*: permutation *p*-*values* computed from the *p*-*value* reported by the OMSSA for the matches. *E*-*value*: permutation *p*-*values* computed using OMSSA *E*-*values*.

c Significance threshold (t) for matched to be considered significant at *p*-*value*<1×10^−t^.

d Incorrect: the program provided an incorrect match.

e Cumulative number of peptides with *p*-*value*<1×10^−2^.

f Cumulative number of peptides with *p*-*value*<1×10^−4^.

Detection and significance computation using the number of matched ions, OMSSA *p-value* and *E-value* indicators was identical across all k-permuted decoy databases. However, the lambda parameter was less suitable than the other OMSSA match indicator to discriminate matches than the other match indicators. Differences in the lambda indicator for the same observed spectrum were mainly determined by the total number of theoretical *m/z* values for product ions and hence by the length of the decoy peptide sequence. After a relatively few permutations, the range of possible sequences is determined such that fewer permutations are required to determine the distribution of the lambda parameter than other match indicators.

### Impact of PTM on Peptide Identification using a K-Permuted Decoy Database

Searches of 80 peptides with no PTMs including the specification of common neuropeptide PTMs improved the significance of the detection in target-k-permuted decoy databases. Using X! Tandem, all 80 observed peptides were identified at *p-value* < 1×10^−5^ using the number of matched ions and hyperscore indicators in the K10^5^ permuted database, while convolution score indicator detected only 7 (8.75% of unmodified neuropeptides) peptides. Consistent with searches without PTMs using the OMSSA program, when the searches included PTMs the number of matched ions and *E-value* indicators provided more significant permutation *p-values* than the lambda indicator. For Crux, specification of PTMs reduced the performance (significance levels) of the number of matched ions, XCorr, and Sp indicators in the K10^5^ database. The lower significances was due to corresponding increase in the decoy peptides with equal or better scores than the target peptides with increase in decoy database size when PTMs are considered in the search. Using the K10^5^ permuted database, OMSSA and X! Tandem correctly identified the 20 and 17 of spectrum with PTMs as the first match, respectively. Both programs correctly identified the same 16 peptides, 6 peptides were identified by only one program and 1 peptide was not detected by either program. There were 4 peptides unmatched by X! Tandem only and the unmodified forms were matched outside the top 20 matches. The unmatched peptide, acetyl-YGGFMTSEKSQTPLVT, was undetected by OMSSA both in the target or k-permuted databases. X! Tandem was able to match the correct sequence, however the match has an additional amidation. Manual evaluation would have corrected the match as the amidation was on an unexpected amino acid and the non-amidated form was closer to the precursor mass then the amidated form.

The remaining 2 peptides that were unmatched by OMSSA were both amidated. One peptide, SYSMEHFRWGKPV-amide, was correctly identified as the 15^th^ best match by OMSSA with the unamidated form providing the best match. The difference in monoisotopic mass between modified and unmodified was less than 1 Da. The experimental spectrum had a precursor *m/z* value of 541.70 with an assigned a 3+ charge state. At a 3+ charge state the predicted *m/z* values were 541.9294 and 541.6014 for the unmodified form and amidated forms, respectively. Biologically the unmodified form would be identified as a probable match since this sequence is an intermediate in the amidation process and the unmodified sequence is uncommon among neuropeptides because this form lacks the terminal G-residue after cleavage [11]. Consequently this unmodified peptide could be considered a match for OMSSA.

### Comparison of Peptide Database Search Programs

Overall the k-permuted decoy databases allowed the detection of more peptides based on real spectra than the use of the standard target database regardless of the database search program. The search of spectra against the k-permuted decoy databases produced many matches that were indistinguishable from each other based on the indicators reported by the programs (e.g., number of matched ions, hyperscore, convolution score, and *E-value* for the X! Tandem). Permutation testing is computational demanding even with Monte Carlo sampling (Table 6). The increase in time across permutated database sizes is a consequence of the exponential increase in the number of sequences evaluated. However, the K10^5^ database provided adequate results and all programs completed the search within 35 CPU minutes using a single process Intel Core i7-3770 CPU @ 3.40 GHz. This timing is the result of single-processor searches that ignored possible parallel processing of individual spectra. The advantages of Monte Carlo permutation approaches to assess the statistical significance of neuropeptide matches could be further advanced by simultaneously running groups of observed spectra using parallel processing.

**Table 6:** Computation times in seconds for search of 80 unmodified spectra against different databases using a single process Intel Core i7-3770 CPU @ 3.40 GHz.

Database^a^	Database Search Program		
	Crux	OMSSA	X! Tandem
Target	5	11	1
K10^3^	7	56	41
K10^4^	61	915	476
K10^5^	200	1220	467
K10^6^	2162	24475	5196

a Target: database of 236 neuropeptide sequences; K10^3^: k-permuted decoy database size of 236,000 peptides; K10^4^: k-permuted decoy database size = 2,360,000 peptides; K10^5^: k-permuted decoy database size = 23,600,000 peptides; K10^6^: k-permuted decoy database size = 236,000,000 peptides.

An alternative approach to generate a permutated database is to perform targeted permutation of specific regions such as the terminal amino acids to disrupt *b-* and *y*-ion series. While other regions can be permuted, the advantage of permuting only the terminal peptides is that this strategy is independent of peptide size. The size of the required database quickly increases from 84,960 sequences per target peptide when one terminal position was permuted to 47,045,880 sequences per target peptide when 3 terminal positions were permuted. Evaluation of terminal permuted databases demonstrated that this approach offered similar yet less significant matches than the whole sequence permuted database approach. Also, this permutation approach had the disadvantage of providing a large number of homeometric matches since experimental ions near the termini are required to differentiate the order of amino acids. Thus, results from this approach are not reported.

With the goal of accurate significance evaluation of protein matches, dynamic programming-related approaches have been proposed [31]. However, dynamic programming assumes that a problem (i.e., spectra matching) can be divided into independent components. In the context of tandem spectra, any division based on sequence location creates dependent components because changing an amino acid in any location will change both the *b*- and *y*-ion fragment series. Further any mass change must be balanced by a corresponding change in another part of the sequence such that the overall mass is within the specified tolerance of the original mass. Also, the implementation of these approaches limit high computational requirements by limiting the information considered or through analytical assumptions. These strategies resulted in non-exhaustive libraries that could lead to biased statistical significance assessment. In one case, the algorithm used is location based such that the only one ion series can be used [16], [17] due to interrelationship between ion series and that precursor must remain within the preset tolerances. However, using only one series is not as effective as using both ion series and that one ion series can be more informative than the other series [17]. In the other case, the score for a given number of matched peaks is assumed to encompass the score from fewer matched peaks [11]. This assumption fails when different sets of peaks are being matched from the same peptide and the number of peaks in common changes. Both strategies do not consider the optimal starting location such that a peptide will be dropped from consideration when a region of the spectrum has a poor match score despite the higher score in other unevaluated regions. The published algorithms appear to lack error corrections for common problems of incorrect peak assigned due to charge state, presence of chimeric peptides, and missing peaks. Also, both dynamic programming strategies do not have a clear approach to account for peptide length that has been proven to bias the statistical significance of neuropeptides identifications [16]. Lastly, both approaches cannot be directly applied to the open source X! Tandem, Crux and OMSSA unlike the straightforward permutation approach proposed in this study. Although the lack of comparable basis challenges the benchmarking of strategies, the Monte Carlo permuted database approach proposed addresses the previous limitations while enabling simple integration to database search programs and prompt results.

## Conclusions

The present study demonstrated that the k-permuted decoy database is an effective and computationally feasible approach to accurately calculate the statistics of neuropeptide matches from complex tandem MS datasets. Unlike other proposed methods to control multiple testing such as target-decoy approaches, permutation testing provided strong control of Type I error such that neuropeptides are detected at high confidence of significance. The implication of this finding is that an extensive decoy database is not required to accurately detect neuropeptides and this can be exploited to increase the speed of the search.

This study demonstrated the relative superiority of specific detection indicators for database search programs. The indicators *E-value*, hyperscore, and Sp score from the OMSSA, X! Tandem, and Crux programs, respectively, performed better than other indicators. The results indicated that 10^5^ permutations per peptide were sufficient to provide significant peptide identifications. Indication of the suitability of the Monte Carlo permutation approach using 10^5^ permutations was the capability of all three database search programs to detect all or nearly all neuropeptides at *p-value* < 10^−4^ and the absence of a trend for lower statistical significance with higher permutation number. A promising finding is the robust performance of the simple indicator, number of matched ions between the experimental and theoretical spectra to detect peptides. This intuitive indicator identified the vast majority of the peptides also identified by other indicators such as hyperscore, Sp and *E-value* that rely on assumptions or parametric specifications. This result also highlights the importance of considering the number of matched ions when a match is borderline significant. The results have shown that, in conjunction with database search programs, the k-permuted sequence databases allowed the detection of more peptides and exhibited high consensus among the various indicators and database search programs.

Permutation testing approached developed here can easily be integrated into standard database search programs to compute spectrum specific *p-values* for any indicator reported by the program. Through the generation of decoy peptides, the permutation approach could offer insights into unknown or unexpected neuropeptides (including those resulting from PTMs or polymorphisms or chimeras) not present in the target database. Further, the k-permuted databases can be generated once and shared between programs and the community.
